# Isolation and Enrichment of Mouse Female Germ Line Stem Cells

**DOI:** 10.22074/cellj.2015.487

**Published:** 2015-01-13

**Authors:** Somayeh Khosravi-Farsani, Fardin Amidi, Mehryar Habibi Roudkenar, Aligholi Sobhani

**Affiliations:** 1 Department of Anatomy, School of Medicine, Tehran University of Medical Sciences, Tehran, Iran; 2 Cellular and Molecular Research Center, Shahrekord University of Medical Sciences, Shahrekord, Iran; 3 Blood Transfusion Research Center, High Institute for Research and Education in Transfusion Medicine, Tehran, Iran

**Keywords:** Stem Cell, Mouse, Ovary, Culture, Enrichment

## Abstract

**Objective:**

The existence of female germ-line stem cells (FGSCs) has been the subject of a wide range of recent studies. Successful isolation and culture of FGSCs could facilitate studies on regenerative medicine and infertility treatments in the near future. Our aim in the present study was evaluation of the most commonly used techniques in enrichment of FGSCs and in establishment of the best procedure.

**Materials and Methods:**

In this experimental study, after digesting neonate ovary from C57Bl/6 mice, we performed 2 different isolation experiments: magnetic activated cell sorting (MACS) and pre-plating. MACS was applied using two different antibodies against mouse vasa homolog (MVH) and stage-specific embryonic antigen-1 (SSEA1) markers. After the cells were passaged and proliferated *in vitro*, colony-forming cells were characterized using reverse transcription-polymerase chain reaction (RT-PCR) (for analysis of expression of *Oct4, Nanog, C-kit, Fragilis, Mvh, Dazl, Scp3 and Zp3*), alkaline phosphatase (AP) activity test and immunocytochemistry.

**Results:**

Data showed that colonies can be seen more frequently in pre-plating technique than that in MACS. Using the SSEA1 antibody with MACS, 1.98 ± 0.49% (Mean ± SDV) positive cells were yield as compared to the total cells sorted. The colonies formed after pre-plating expressed pluripotency and germ stem cell markers (*Oct4, Nanog, C-kit, Fragilis, Mvh* and *Dazl*) whereas did not express *Zp3* and *Scp3* at the mRNA level. Immunocytochemistry in these colonies further confirmed the presence of OCT4 and MVH proteins, and AP activity measured by AP-kit showed positive reaction.

**Conclusion:**

We established a simple and an efficient pre-plating technique to culture and to enrich FGSCs from neonatal mouse ovaries.

## Introduction

Until recently, it has been generally accepted that all female germ cells commit to meiosis entrance before birth (1-3). Recent findings indicate that adult ovaries may have pluripotent cells (4-7). This specialized cell population is referred to as female germ-line stem cells (FGSCs) (5). Researchers could isolate these stem cells successfully from neonate and adult mice, while the cells were shown to expand *in vitro* for months (6-8). In one of the experiments after transplantation of green fluorescent protein (GFP)-expressing isolated FGSCs to sterilized mice, cells could differentiate to mature oocytes, leading to GFP positive offspring (6). Recently White et al. (8) showed that isolated FGSCs from adult mouse ovaries and human ovarian cortical tissues have potential to expand *in vitro* and could spontaneously generate 35 to 50 mm oocytes. This group also showed that injection of the human germ-line cells, expressing GFP, into human ovarian cortical biopsies leads to formation of follicles containing GFP-positive oocytes after xenotransplantation into immunodeficient female mice. The authors proposed that immature germ cells present in the adult mammals may be able to generate de novo oocytes in a favorable environment. As Tilly and Telfer stated, by repopulating adult ovaries with new oocytes and follicles, clinical intervention would no longer be an unreachable target (9). For establishing stem cells from mouse ovaries, researchers employed different techniques. They included magnetic activated cell sorting (MACS) using immunomagnetic beads coupled to mouse vasa homolog (MVH) and stage-specific embryonic antigen-1 (SSEA1), fluorescence- activated cell sorting (FACS) by MVH antibody, a transgenic mouse model in which GFP was expressed under a germ cell-specific Oct4 promoter and pre-plating (4, 6-8, 10). In these studies, they used neonate (6, 7) or adult ovarian tissues (4, 6, 8, 10). Although this body of work represents an important advance in the field of stem cell and neo-oogenesis, more studies are needed to be done to extend their finding in developing an effective strategy to purify and to enrich FGSCs. In this study, we tried to enrich these FGSCs by immunomagnetic isolation using MVH and SSEA1 antibodies and also by morphology-based selection after pre-plating technique. The latter has been used for enrichment of male germ-line stem cells (MGSCs) from testis (11). As researchers have found that FGSCs will decline by advancing age (12, 13), neonate ovaries were selected for our experiments. Then, we compared the colony formation in 3 different experiments, and finally, we characterized colonies and defined the potential for proliferation *in vitro* in the best obtained results.

## Materials and Methods

All the chemicals used in this experimental study, except those mentioned, were purchased from Sigma- Aldrich Chemie, Germany. The Ethic Committee of Tehran University of Medical Sciences confirmed the study.

### Mouse embryonic fibroblast (MEF) cell preparation

Embryos (E13-16) from a pregnant C57Bl/6 mouse were removed and rinsed in phosphatebuffered saline (PBS). The placenta and fetal membranes, head, liver and heart were removed. Mouse embryonic fibroblast (MEF) cells suspension were collected after tissue digestion in 0.25% trypsin solution and passed through a screen. Cells were cultured in Dulbecco’s modified Eagle’s medium (DMEM) with high-glucose (Gibco, USA), 1% non-essential amino acids (Gibco, USA), 10% fetal bovine serum (FBS) (Gibco, USA), 1% glutamax (Gibco, USA), and penicillin/ streptomycin (Gibco, USA). We used MEF passages 2-4 times to make feeder layer. A density of 5×10^4^ inactivated MEF cells/ cm (treated with 10 μg/ml mitomycin C) was suitable as feeder layer for cultivation of undifferentiated FGSCs.

### Ovarian cell preparation

We used about 20 ovaries from 3- to 5-dayold C57Bl/6 mice for each experiment. For isolation of ovarian cells, an enzymatic digestion method was used as described previously (7). Briefly, after complete dispersion in 1 mg/ml collagenase type IV (Gibco, USA) and DNase type I (10 μg/ml), enzyme was neutralized by adding 10% FBS. The dissociated cells were passed through a 30 μm cell strainer (130-041- 407, MiltenyiBiotec Inc., UK). The cell suspension was centrifuged at 300 g for 5 minutes, the supernatant was discarded, and the pellet was then subjected to 3 different experiments. The animal care was conducted in accordance with the institutional guidelines of Tehran University of Medical Sciences and the National Institutes of Health (NIH) guidelines for the care and use of laboratory animals.

### Isolation and purification of FGSCs

#### Experimental design

##### Experiment 1: isolation of SSEA1^+^ cells by MACS

SSEA1 is supposed to be expressed on mouse primordial germ cells (PGCs) and mouse stem cells (14, 15). Therefore, ovarian cell pellet was re-suspended in MACS buffer [PBS containing 0.5% BSA and 2 mM ethylene diaminetetraacetic acid (EDTA)]. Anti-SSEA1 microbead (130-094-530, MiltenyiBiotec Inc., UK) was added to the cell suspension, and SSEA1^+^ cells were then separated on MS columns in a mini MACS separation unit according to the manufacturerʼs instruction (MiltenyiBiotec Inc., UK). Isolated cells were transferred onto a mitotically inactivated MEF in 4-well plates containing culture media as described previously (6) at 37˚C in a 5% CO_2_ atmosphere.

##### Flow cytometry

Purity of the separated population just after MACS was determined by flow cytometry according to the supplier’s recommendations. Briefly, positively SSEA1 selected population was diluted in 100 μl of MACS buffer and incubated with 10 μl of anti-SSEA1/phycoerythrin (PE)-conjugated antibody (130-091-375, MiltenyiBiotec Inc., UK) for 20 minutes in the dark (refrigerator). This antibody can selectively bind to SSEA1 marker on the cell surface, and fluorescent dye conjugated to the antibody will tag SSEA1 positive cell. Cells were then centrifuged, washed and re-suspended in PBS just before analysis.

##### Experiment 2: isolation of MVH+ cells by MACS

The pellet of ovarian cells was re-suspended and incubated with polyclonal rabbit antimouse MVH (ab13840, Abcam, Cambridge, UK) and subsequently by goat anti-rabbit IgG magnetic beads (130-048-602, MiltenyiBiotec Inc., UK). The mixture of cells and magnetic beads was placed on the magnetic bead separator, while separation was performed following the manufacturerʼs instructions and as described in experiment 1. Isolated cells were cultured on mitotically inactivated MEF in 4 -well plates at 37˚C in a 5% CO_2_ atmosphere.

##### Experiment 3: isolation based on morphology selection after pre-plating

The pre-plate technique is based on the differential adherence characteristics of primary culture cells to a gelatin-coated surface. The pellet was re-suspended in culture medium and subsequently pre-cultured onto a 60×15 mm gelatinized culture dish. As Gong et al. (10) stated, due to the different adherence rates, the somatic cells attached quickly to the bottom of the dishes and the supernatant was withdrawn after 45 minutes. Cells were re-plated onto a mitotically inactivated MEF monolayer in a 35 mm dish. The cultures were passaged 2 times before they were subjected in characterization.

#### Culture of ovarian cell

Ovarian cells obtained from 3 mentioned experiments were cultured at a density of 2×10^3^ ml^-1^ on MEF feeders in culture medium. The culture medium was α-Minimum essential medium (α-MEM) containing 0.1 mM ß-mercaptoethanol, 10% FBS, 1% nonessential amino acids (Gibco, USA), 1 mM sodium pyruvate, 1% lyophilized mixture of penicillin and streptomycin (Gibco, USA), 1000 units/ mL mouse leukemia inhibitory factor (LIF), 2 mM L-glutamine, 10 ng/ml epidermal growth factor (EGF), 50 ng/ml human glial cell line-derived neurotrophic factor (GDNF) (R&D, UK), and 1ng/ml human basic fibroblast growth factor (bFGF) (BD Biosciences, UK) at 37˚C in a 5% CO_2_ atmosphere. After 7-10 days, colonies were mechanically split 1:2 and placed on a new prepared MEF monolayer. The medium was changed every other day and cultures were passaged every week.

#### Characterization of FGSCs

Analysis of transcription markers, including Nanog, C-kit, Oct-4, Fragilis, Mvh, Dazl, Zp3 and Scp3, expression levels by reverse transcription-polymerase chain reaction (RT-PCR)

A total of 15-20 colonies were picked up mechanically and total RNA was extracted from each sample using RNX-Plus^TM^ kit (RN7713C, CinnaGenCo., Iran) according to the manufacturer's recommendations. cDNA was synthesized using PrimeScript^TM^ RT reagent kit by the manufacturer's protocol (Ta- KaRa, Japan) and the PCR was performed with HotStarTag DNA Polymerase (Qiagen, Germany) for each primer (Table 1). General reaction conditions were 95°C for 5 minutes followed by 35 cycles at 95°C for 45 seconds, the appropriate annealing temperature for 30 seconds, elongation by heating at 72°C for 35 seconds and a final extension at 72°C for 10 minutes. The Gapdh gene was used as expression control and a non-template control (No RT, without cDNA) was included in each run.

**Table 1:** Primers for determination of mice markers using reverse transcription-polymerase chain reaction (RT-PCR)


Gene	Primer sequence (5´- 3´)	Product size (bp)	Accession number

**c-Kit**	F: CTCACATAGCAGGGAGCACA	123	NM_001122733.1
	R: ACAACTCACCCACACGCATA		
**Nanog**	F: CTGCTCCGCTCCATAACTTC	97	NM_028016.2
	R: GCTTCCAAATTCACCTCCAA		
**Fragilis**	F: AGCCTATGCCTACTCCGTGA	112	NM_025378.2
	R: GAGGACCAAGGTGCTGATGT		
**Oct4**	F: GTTCTCTTTGGAAAGGTG	147	NM_001252452.1
	R: GCATATCTCCTGAAGGTT		
**Mvh**	F: GCTCAAACAGGGTCTGGGAAG	145	NM_010029.2
	R: GCTTGATCAGTTCTCGAG		
**Scp3**	F: CCGCTGAGCAAACATCTAAAGATG	161	NM_011517.2
	R: GGAGCCTTTTCATCAGCAACAT		
**Dazl**	F: AAGGCAAAATCATGCCAAAC	72	NM_010021.4
	R: TCCTGATTTCGGTTTCATCC		
**Zp3**	F: TAACCGTGTGGAGGTACCCA	136	NM_011776.1
	R: CAGGCGAAGAGAGAAAGCCA		
**Gapdh**	F: ACACCCACTCCTCCACCTTTG	112	NM_002046.4
	R: TCCACCACCCTGTTGCTGTAG		


### Immunocytochemistry assay of OCT-4 and MVH markers in the ovarian cell culture

Immunocytochemistry was performed to determine whether these ovarian cells express FGSC markers, OCT-4 and MVH. OCT-4 is a pluripotency and early germ cell development marker (16) and MVH is a germ-line marker (17, 18). The colonies grown on the glass slides were stained. Briefly, cells were fixed by paraformaldehyde 4% for 15 minutes at room temperature (RT) and then permeabilized with 0.1% triton-X for 15 minutes at RT. Cells were washed and blocked in 10% normal goat serum in PBS at RT for 60 minutes and incubated overnight at 4˚C with primary antibodies of MVH (Dilution: 1: 100; Ab13840) and OCT4 (Dilution: 1: 200; Ab18976) that were diluted in blocking solution. For negative control, the primary antibody was omitted. After washing with PBS, cells were incubated with secondary antibody (Ab 6717) for 1 hour at RT. Then culture was incubated with 4´, 6-diamidino-2-phenylindole (DAPI, D8417) for 10 minutes at RT. Images were captured using a fluorescent microscope.

### Alkaline phosphatase (AP) activity analysis

For assessing the AP-activity, the cells were fixed with 4% paraformaldehyde and stained histochemically using an AP-staining kit (F4523) following the manufacturerʼs protocol.

### Statistical analysis

Each experiment was repeated at least five times. Statistical analysis after flow cytometry was performed using the Statistical Package for the Social Sciences (SPSS, SPSS Inc., Chicago, IL, USA) version 16.0 and the significance was attributed at p≤0.05.

## Results

### Experiment 1

**Fig 1:**
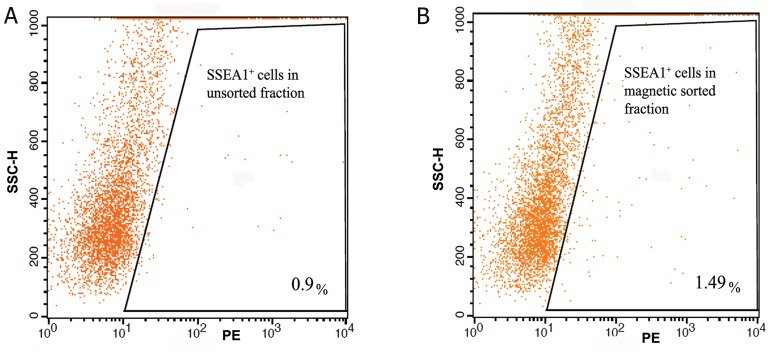
Flow cytometric analysis of PE signal after MACS of neonate ovarian cells using an SSEA1 antibody. Figures show the relative number of PE-labeled cells within unsorted (A) and magnetic sorted (B) fractions. The enrichment of PE-positive cells (conjugated with SSEA1 antibody) after MACS is ignorable. PE; Phycoerythrin, MACS; Magnetic activated cell sorting, SSEA1; Stage-specific embryonic antigen-1 and SSC-H; Side light scatter

As a result of first experiment, positive selection of female germ line stem cell by MACS did not increase the percentage of these cells as it was shown by the number of colonies formed after culture *in vitro* and flow cytometry. Isolated cells could form 1-2 colonies per well after subsequent culture on MEF feeder layer. Flow cytometry was used to analyze the relative number of SSEA1^+^ PE-labeled cells in magnetic fraction obtained (Fig 1). The sorted cell fractions contained about 1.98 ± 0.49% (Mean ± SDV) PE-positive cells in flow cytometry analysis.

### Experiment 2

After using MVH-magnetic bead sorting for the enrichment of FGSCs, isolated cells could form some colonies after subsequent culture on MEF feeder layer. The number of colonies was obviously lower than that in pre-plating experiment (again 1-2 colonies per well in 4-well plates). The advantage of this experiment was reducing the number of fibroblast cells.

### Experiment 3

**Fig 2:**
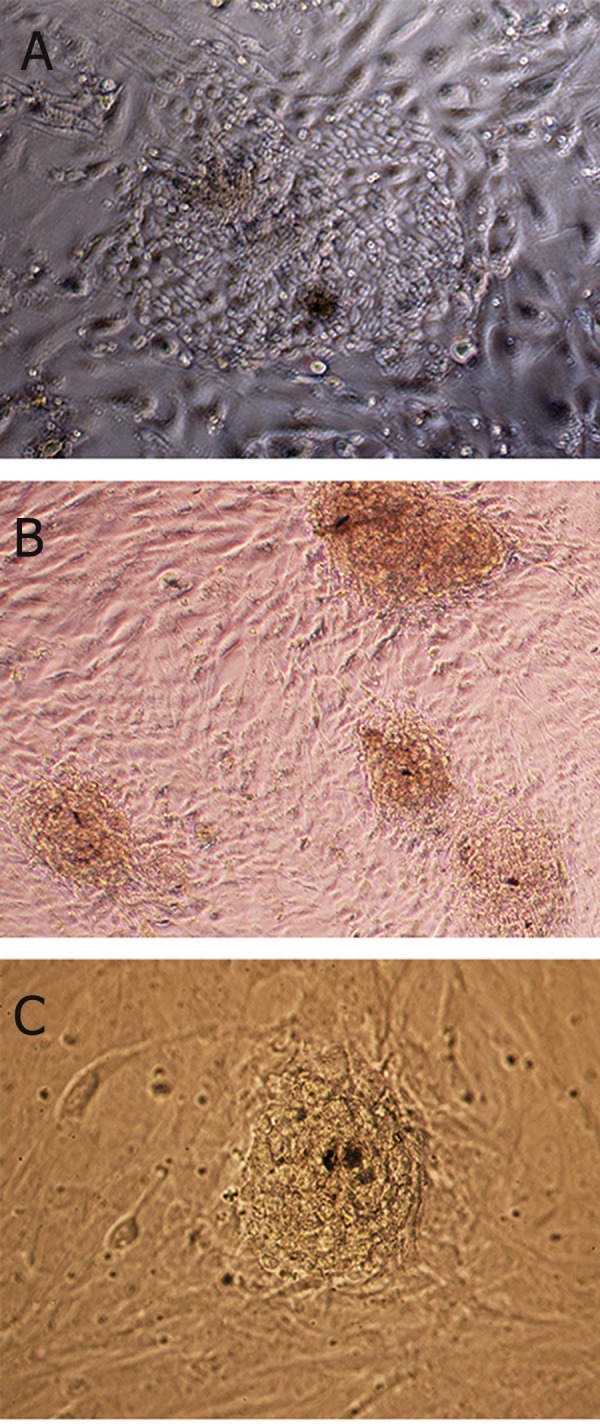
Morphology of colonies cultured after pre-plating technique. A. Loosely associated colonies observed on day 3 of culture that grown as loose monolayer cluster (×20). B, C. Tightly associated colonies at the end of first week [×10(B) and ×20(C)].

As shown in figure 2, when pre-plating technique was used for enrichment of FGSCs, the cells increased in number and formed 10-12 small colonies per 35 mm dish after one week of culture. Two markedly different forms of colonies were observed: type A, appeared after day 2 of culture and grown as large monolayer colonies (Fig 2A), while type B, appeared after 1 week of culture and grown as small compact colonies (Fig 2B, C). Latter colonies were successfully passaged mechanically in our laboratory. Based on our experiences, growth factors in the culture media seemed to be necessary as without them, colony formation was lower and cells began to die in a shorter time. It may show the importance of these growth factors in proliferation and self-renewal of FGSC. In this experiment, after second passage, colonies in culture were picked up for characterizing assays.

### Analysis of transcription markers by RT-PCR

**Fig 3:**
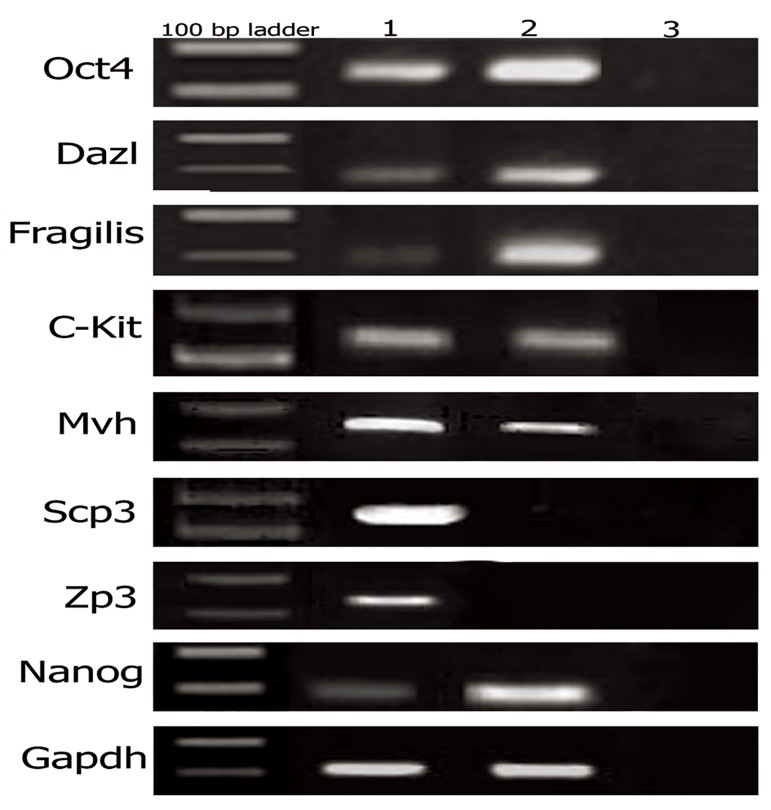
RT-PCR analysis of expression of C-kit, Nanog, Oct4, Fragilis, Dazl, Mvh, Scp3 and Zp3 in ovary (lane 1), FGSCs (lane 2) and no RT-PCR analysis (lane 3). Gapdh mRNA expression was used as an internal control. FGSCs; Female germ-line stem cells.

RT-PCR was performed on extracted mRNA from mouse neonate ovary and the FGSC small colony (after passage 2). Ovaries and FGSC colonies were positive for mRNA expression of *Oct4, Nanog, C-kit, Mvh, Dazl* and *Fragilis*. As expected, no bands were detected for *Scp3* and *Zp3* in FGSC colonies, suggesting that these colonies failed to enter to meiosis (Fig 3).

### Characterization of ovarian FGSCs by immunocytochemistry

**Fig 4:**
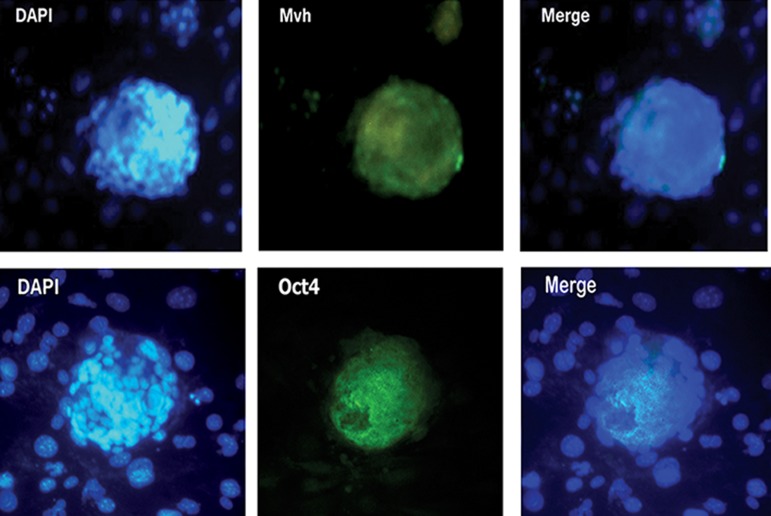
Immunocytochemistry of FGSCs colonies using antibodies (FITC-conjugated secondary antibody, green) against the germ-line markers MVH (top) and OCT4 (bottom). Nuclei were stained with DAPI. As shown by fluorescence microscopy, FGSCs expressed the markers MVH and OCT4 (×20). FGSCs; Female germ-line stem cells, FITC; Fluorescein isothiocyanate, MVH; Mouse vasa homolog and DAPI; 4', 6-diamidino- 2-phenylindole.

Immunocytochemical staining with anti-MVH and anti-OCT4 antibodies further confirmed that cells within the colonies are expressing FGSC proteins (Fig 4).

### AP activity

**Fig 5:**
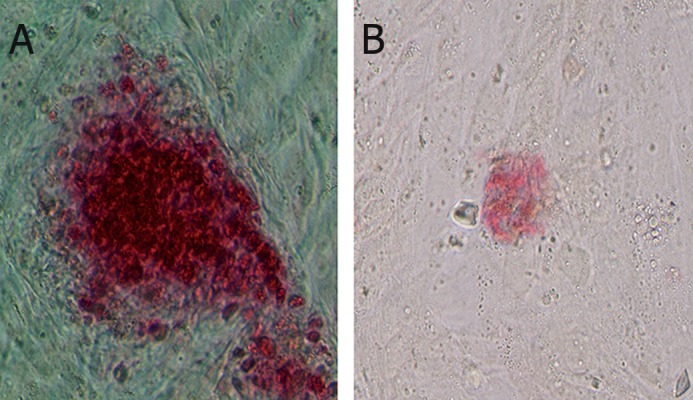
AP-positive cells (red) after second passage. Notethatlevels of staining intensity within culture are from strong in A (×20) to weak reaction in B (×20). AP; Alkaline phosphatase.

Compact colonies formed in culture were positive for AP activity (Fig 5). As seen, the staining intensity was variable. Some of them had strong reaction, like embryonic stem cells, whereas some of them had weak reaction. High levels of AP activity were associated with multicellular colonies in culture.

## Discussion

The present study compared three procedures for enriching FGSCs from digested ovarian tissues. By culturing ovarian cells, we could demonstrate that 2 distinct types of colonies will form which are distinguished based on their morphology (Fig 2). The first type appeared around day 2 and grown as monolayer cluster. Although RT-PCR showed that they express *Oct4* slightly, they did not react in AP staining and also did not express germ cell markers (data not shown). The morphology was similar to what Gong et al. (10) reported as follicular cells. They showed that these cells have strong reactivity with anti-mullerian hormone (AMH) that is an ovarian follicular somatic cell marker. We observed the second type of cells as compact colonies after 1 week of culture of ovarian cells. RT-PCR showed that the cells express *Oct4, Nanog, Mvh* (pluripotent gene transcripts), *Dazl, Fragilis* (germ cell markers), *c-kit* (stem cell factor receptor), but did not express *Scp3* and *ZP3*. Expression presented here was consistent with Zou et al. (6) who isolated stem cells that were positive for germ cell markers. However colony forming cells in their study were not expressing *Nanog* and *ckit*. It has been found that *c-kit* is involved in survival and proliferation of migrating mouse PGC that is found in early germ cells and in most stem cells (19). Gong et al. (10) have reported embryonic stem (ES) cell-like colonies by co-culture of ovarian cells on an inactivated MEF monolayer that were expressing ES gens (showed in RT-PCR and immunostaining) but not markers of germ line (*Mvh* and *Fragilis*) after immunostaining. In our study, *Mvh* gene was expressed at the levels of RNA (after RT-PCR) and protein (after immunostaining). In addition, these compact colonies in our study were positive for AP. Evidence of AP expression in these cells suggests that they are not undergoing meiosis (19, 20). Collectively, expressing PGC markers show that cells are probably in a transitional stage of their development from ES cells (from unknown source), or they are some PGC derived stem cells. De Felici suggested that probably a small number of PGC/oogoniaor PGC derived undifferentiated cells with germ stem cell (GSC) characteristics remains in the post-natal and adult ovary and under certain conditions may resume mitosis, enter meiosis and give rise to oocytes (12). Parte et al. (21) stated that the main advantage of being in quiescent nature is protection from accumulation of chromosomal aberrations associated with the process of aging.

Attempts to enrich MVH+ cells and SSEA1^+^ cells in our study by MACS were unsuccessful. The possibility cannot be excluded that the enzymatic digestion during preparation of the single-cell suspension changes epitopes at the outer plasma membrane (22), or primary antibody is unable to detect receptor protein on the surface of germ cell, especially about MVH receptor that is widely considered to be a cytoplasmic protein. However, stem cell colonies formed in culture after pre-plating were labeled for MVH after immunostaining, ensuring cytoplasmic expression of this marker. It has been stated that MVH is a general germ cell marker and does not enrich germ line stem cells in the ovary (7). So immunomagnetic separation may not be as efficient and needs negative enrichment of target cells by depleting non-target cells (23, 24), while should be consider for FGSC in ovary. It can also cause problems if the cell type of interest does not have unique markers. It has been shown that MVH in ovary is expressed in the germ cells with different developmental stages, while making the isolation of a homogeneous population difficult (7, 24). FGSC separation is a new established field and there are challenges and difficulties to overcome. The detailed knowledge of the biology of FGSCs and the potential techniques is necessary to select the correct methodology to yield the desired cell population. It is noteworthy to mention that it would be expensive doing FACS and MACS with special antibody to have 1.5 ± 0.2% (with MVH antibody in adult mice) (25) or 1.98 ± 0.49% (with SSEA1 antibody in neonate mice in our study) positive cells in sorted population. This purity is low when compared withthe sorted equivalent male population (22). Some Studies have reported *in vitro* differentiation of mouse ESCs into germ cell and presumptive oocytes (26, 27). These finding show that embryonic cells can enter in meiotic process and may give rise to gametes. However, it seems that *in vivo* gametogenesis and final maturation with oocyte production is happening in a complex niche that is controlled by signals from somatic cells (28). Some studies have demonstrated that proliferative capacity of stem cells to respond to signal diminish with advancing age (29, 30). This delicate network remains to be determined. Further investigation should be focused in improving stem cell isolation methods from ovary in order to be used in *in vitro* fertilization (IVF) clinic for subsequent differentiation and also in regenerative medicine by determining what is happening in the ovarian complex network. This will provide information in rejuvenating of ovary to a younger state and improvements in health and well-being in aging females.

## Conclusion

Pre-plating purification is easy and efficient, while does not require special equipment. Such a simple manipulation promise is important for designing strategies to maximize isolation and enrichment of FGSCs.
